# Copper Ion as a New Leakage Tracer

**Published:** 2013-12

**Authors:** J Modaresi, M Baharizade, A Shareghi, M Ahmadi, A Daneshkazemi

**Affiliations:** a Dept. of Endodontics, School of Dentistry, Shahid Sadoughi University of Medical Sciences, Yazd, Iran; b General Dentist; c Dept. of Endodontics, School of Dentistry, Shahrekord University of Medical Sciences, Shahrekord, Iran; d Post graduate student, Dept. of Endodontics, School of Dentistry, Shahid Sadoughi University of Medical Sciences, Yazd, Iran; e Dept. of Restorative, School of Dentistry, Shahid Sadoughi University of Medical Sciences, Yazd, Iran

**Keywords:** Apical Leakage, Copper Diffusion, Dye Penetration, Methylene Blue

## Abstract

**Statement of Problem**: Most failures of root canal treatments are caused by bacteria. Studies showed that the most common cause of endodontic failures were the incomplete obturation of the root canal and the lack of adequate apical seal. Some *in-vitro* methods are used to estimate sealing quality, generally by measuring microleakage that allows the tracer agent to penetrate the filled canal.

**Purpose: **Conventional methods of evaluating the seal of endodontically treated teeth are complicated and have some drawbacks. We used copper ion diffusion method to assess the leakage and the results were compared to dye penetration method.

**Materials and Method: **The crowns of 21 extracted teeth were cut off at the CEJ level. After preparing the canals, the teeth were placed in tubes containing saline. They were divided randomly into 15 experimental cases; 3 positive and 3 negative controls. Positive controls were filled by single cone without sealer while the experimental and the negative control groups were filled by lateral technique. The coronal portion of gutta was removed and 9mm was left. The external surface of each tooth was coated with nail polish. Two millimeters of apical portion was immersed into 9ml of distilled water and 0.3ml of CuSO4 solution was injected into the coronal portion. After 2 days, copper sulfate was measured by an atomic absorption spectrophotometer. The teeth were then immersed in 2% methylene blue for 24 hours, sectioned and the extent of dye penetration was measured by a stereomicroscope.

**Results:** The maximum and minimum recorded copper ion concentrations for the experimental group were 18.37 and 2.87ppm respectively. The maximum and minimum recorded dye penetrations for the experimental group were 8.5 and 3.5mm respectively. The statistical analysis, adopting paired samples test, showed poor correlation between average recorded results of two methods.

**Conclusion:** Based on our results, there was no significant correlation between the dye penetration and the copper ion diffusion methods.

## Introduction

Most failures of root canal treatment are directly or indirectly caused by bacteria that remain active in the dentinal tubules even after vigorous chemo-mechanical preparation [1]. So the aim of obturation is to create a complete seal along the length of root canal system from the coronal opening to the apical termination [2]. Perfect apical seal is desirable to prevent remaining bacteria and their endotoxins from reaching to the apex [3]. Studies showed that the most common cause of endodontic failures were incomplete obturation of the root canal and lack of adequate apical seal [4]. 

Some *in vitro *methods are used to evaluate the sealing quality, generally by measuring micro-leakage. The traceable agent penetrates through the interface between filling material and canal walls and then it will be traced. Tracers that are used commonly include dyes, bacteria and their products such as endotoxins. Other methods such as fluid filtration, electrochemical techni-que, scanning electron microscopy and radioisotopes assay have also been used [5- 9]. 

The dye leakage evaluation is the most commonly used technique, probably due to its simplicity for evaluation of the leakage. This is a passive method and depends on the capillary fluid movement. Despite the widespread use of this method for evaluating leakage, it may have some limitations. If not controlled precisely, the probability of error findings may increase in this method. Some variables could significantly change the results and quantitative measurements could not be obtained [10].

 In the electrochemical method, the ionic elements and voids in tooth structure may interfere with electrical flow and subsequent leakage measurement. Besides, the intermediate medium (electrolyte) that is used may affect the results [11]. The electrochemical method is based on the electrical resistance and depends on electrical laws. Another disadvantage of this method was its sensitivity since the electrical transmission of materials change with time due to the continuous setting reaction of the materials. Additionally quantitative and qualitative analysis is difficult [11].

The bacterial method is complicated as cultivating and controlling the bacterial population is difficult. It doesn’t have standard models and the reproducibility and comparing the results would be a problem [11]. This method is purely qualitative and can only display the gaps in which bacteria can pass through. It does not reflect the smaller gaps which can be accessed by fluid flow such as ions, toxins and bacterial by products [12].

The radioisotope penetration method is a qualitative method. In the analysis of results; its two-dimensional autoradiograph image is not representative of the three-dimensional image of micro-leakage. An isotope, such as Ca, has an affinity to tooth structure or restorative materials leading to increased measurement errors. In addition, isotopes are able to pass through tooth structure or restorative flaws because of their tiny size; resulting in misinterpretation of leakage [13]. 

Most conventional methods are quite complicated and have some limitations. Hence, in the present study, copper sulfate diffusion method was used since copper ions are not naturally detected in tooth structure by conventional methods. On the other hand, its measure-ment by atomic absorption spectrometer is simple and doesn't interfere with the absorption spectrum of other elements. It seems that this method is highly sensitive and quantitative evaluation of the element is also possible [14]. Thus the purpose of this study was to compare two methods of apical leakage evaluation; dye penetration and copper ion diffusion, to determine the leakage through the root canal fillings and to find the correlation between them if any.

## Materials and Method

Twenty one extracted single root maxillary and mandibular anterior teeth were evaluated in this study. To remove organic debris; the teeth were immersed in 5.25% NaOCl for one hour. The crowns were cut off at the cementoenamel junction with a diamond disk under water coolant. Following pulpal removal, a K-file ISO #10 was inserted into the canal until it reached the anatomical apex. Working length was determined 1mm shorter than that length. The root canals were instrumented using step-back technique to ISO #40 master apical file and the teeth were placed separately in test tubes containing normal saline. 

The roots were randomly divided into one experimental group consisting 15 teeth; 3 positive controls and 3 negative controls. Positive control teeth were obturated only with a single master cone without applying the sealer. Experimental and negative control groups were obturated with gutta-percha and sealer, using lateral condensation technique. The samples were stored in a humidifier for 24 hours to allow the sealer to set. A No.3 Gates Glidden drill was used to remove the coronal portion of the gutta-percha up to 9mm from the apex. The root surfaces in the experimental and positive control groups were coated by two layers of nail polish except for 2mm of the apex and coronal surface. In the negative control group, the entire root surface except the coronal surface was coated with nail polish. Two millimeters of apical portion of the roots were immersed into double distilled water in glassware which was washed by 10% HNO_3 _to eliminate the copper salts. The glassware was filled with 9ml of double distilled water. Then 0.3ml of grlitCuSo_4 _solution (20 gr/lit; Merck, Germany) was injected into the coronal portion of the specimens by a fine needle. After two days, the concentration of the copper sulfate in the solution was measured by atomic absorption spectrometer (Perkin Elmer; Uberlingen, Germany). Then the coronal portion of the root canals were filled with zonalin and kept in room temperature and 100% humidity for 4 hours for setting. Coronal surface of the roots were coated with nail polish. Then teeth were immersed in 2% methylene blue for 24 hours. The teeth were flushed with tab water and longitudinally sectioned with a diamond disk. With the aid of this sectioning method, we examined the exposed filling material since dye penetrates into all spaces between the canal walls and filling materials. The extent of the dye penetration through the canal was measured with a stereomicroscope. For each specimen, the correlation between the atomic absorption readings (copper concentration) and the dye penetration results were evaluated by employing paired samples test.

**Table 1:** The data obtained from copper ion diffusion and dye penetration measurements

**Dye Penetration** **(mm)**	**Copper ion Diffusion** **(ppb)**	**Case no.**
5.5	32	1
5.5	13.78	2
5.5	12	3
3	7.37	4
8.5	7	5
3.5	13.5	6
8	8	7
9	10	8
8	12	9
6.5	5.76	10
8.5	18.38	11
7	7.88	12
3.5	11.96	13
6	9.15	14
5	2.87	15
5	6.49	16
3	9.23	17
5	11	18
0	0	19
0	0	20
0	0	21

## Results

**Figure 1:**
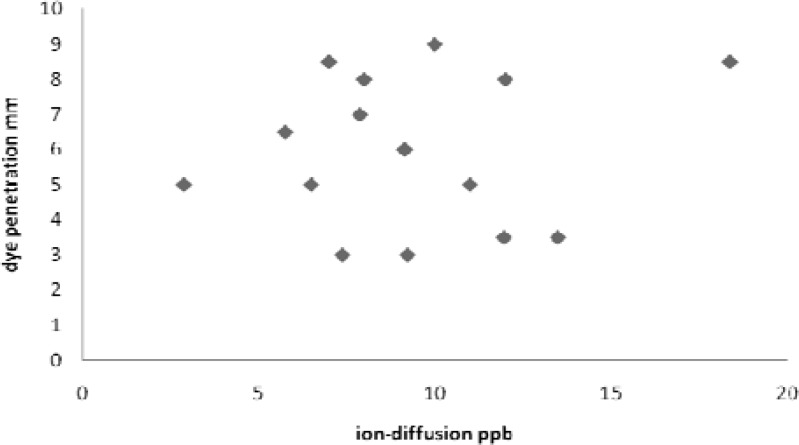
Correlation diagram of leakage in two methods (Scattering diagram)

In negative control teeth, copper sulfate was not detectable. The positive control teeth showed maximum copper sulfate concentration (19.29ppm) and methylene blue dye penetrated in the whole length of gutta-percha. The maximum and minimum recorded copper concentrations for the experimental group were 18.38ppm and 2.87ppm respectively (Table 1). The maximum and minimum recorded dye penetrations for the experimental group were 8.5 mm and 3.5 mm respectively (Table 1). The results of both methods were compared with each other. The statistical analysis, using paired samples test, showed poor correlation between the average recorded results of dye penetration method and the ion diffusion method (r =0.136, *p* =0.63) (Figure 1).

## Discussion

The quality of apical seal with root canal obturation materials has been assessed by various methods such as dye penetration, radioisotope penetration, bacterial leakage, fluorometric and electrochemical means, fluid filtration, scanning electron microscopy and gas chromatography [11]. 

The dye penetration was used because of its simplicity; ease of performance and for not requiring the sophisticated materials. The methylene blue dye was used since its molecular size is similar to bacterial by-products such as butyric acid; which can leak out of infected root canals to irritate periapical tissues [15]. Additionally, this dye is not absorbed by dentin hydroxyapatite crystals. It seems that factors like difference in experimental technique, period of exposure to dye, entrapped air voids and the type of employed tracer likely affect the extent of dye penetration [16]. Particle molecular size, pH and chemical reactivity are anticipated to influence the degree of penetration of the dyes [17]. Different affinity of dyes, to the canal walls and dentin, results in different dye penetrations. 

In electrochemical method, the electrical conductivity is directly related to the concentration of ions in the medium which is proportional to the solubility of ionic material [18-19]. Jorge Luis Goncalves indicated that the concentration of ions in solution increased as the solubility of the sample increased which led to higher conductivity values. Ionic components that release from the sealers may also affect the electrical conductivity [20].

In bacterial method, some sealers with antimicrobial properties (such as silver containing sealers) restrict the penetration of a specific microbe that is used in the study. Consequently, the leakage would be assessed lower than its real value. In ion diffusion method there is not such drawback [11]. 

In this study, copper sulfate diffusion method was used. Naturally, conventional methods do not detect the copper element in tooth structure. Copper ions do not interfere in absorption spectrum with other elements. It seems that this method is very sensitive and can quantitatively evaluate the leakage [14]. Although there are numerous comparing studies on leakage evaluating methods, most of them have shown no significant correlation [5, 9, 16, 25].

 Delivanis and Chapman [21] compared the electrochemical, dye penetration and the radioisotope methods and found correlations in the two extremes of the electric score ranges. Martell and Chandler [22] compared three root-end restorative materials using electrochemical and dye penetration methods and found a correlation between these two methods. However, Matloff et al. [23] reported no correlation between dye penetration and radioisotope methods. Similarly, a study by Barthell et al. showed no correlation between dye penetration and bacterial leakage test methods [6]. Moreover, Pommel et al. [5] found no correlation between the dye penetration, electrochemical and the fluid filtration methods. Chen et al. (1993) compared methylene blue penetration with potassium (KCL solution) diffusion methods. They concluded that there was a positive correlation between them [24]. Camps and Pashly (2003) found that the fluid filtration technique gave results similar to the dye extraction technique, but they did not find a correlation between the classical dye penetration method and the fluid filtration technique [25]. According to the study of Camps and Pashley [25], the reason for this lack of correlation is related to the physical mechanisms involved in these methods. De Gee, Wu and Wessel Ink (1994) and Pommel, Jacquot and Camps (2001), found no correlation between the dye penetration and the fluid filtration techniques [5, 9]. Modaresi (2007) found no correlation between the results of the dye penetration and the electrochemical methods [16]. In most of the studies, no correlation was found between previously introduced methods. Although we may encounter some limitations in this method, the results of this *in vitro* study- similar to many other studies- revealed poor correlation between the dye penetration and the newly designed copper sulfate diffusion method. 

Considering different detection methods in various studies, it is dialectical to achieve these results. Since there was some penetration in experimental group, copper sulfate can be a leakage tracer.

## Conclusion

The obtained results revealed that there was not a significant correlation between the dye penetration and the copper ion diffusion methods. More studies, employing different methods, are necessary to compare the apical seal of various filling materials.
